# The meaning and clinical impact of the protection from harm criterion in Australia’s mental health legislation

**DOI:** 10.1177/00048674251384063

**Published:** 2025-11-06

**Authors:** Christopher James Ryan, Sascha Callaghan

**Affiliations:** 1 Discipline of Psychiatry and Mental Health, University of New South Wales, Sydney, NSW, Australia; 2 School of Medicine, University of Notre Dame, Sydney NSW, Australia; 3 Department of Psychiatry, St. Vincent’s Hospital, Darlinghurst, NSW, Australia; 4 Faculty of Law, University of Technology Sydney, Sydney, NSW, Australia

**Keywords:** Involuntary treatment, mental health legislation, ‘commitment of mentally ill’, clinical practice, psychiatric admission, benefits and harms, medical ethics

## Abstract

This paper examines the legal meaning and clinical impact of the different harm to self and others criteria as they appear in the provisions permitting involuntary inpatient treatment in each of Australia’s mental health acts. The wording of each criterion is reviewed along with relevant Court decisions, explanatory memoranda, second reading speeches and advice published by governments. Each jurisdiction’s harm criterion is set out along with: the breadth of scope of the harms envisaged; how severe any harm must be to trigger the criterion; and how likely it must be that the envisaged harm will arise. The paper is designed so that readers from each jurisdiction may focus on advice relevant to their jurisdiction. In most clinical encounters where involuntary hospitalisation is proposed, the most salient harms for consideration are serious psychological harms and harms to relationships, alongside physical harm to self or others where relevant. The harm criterion sets the minimum level of harm that must be anticipated before clinicians have legal authority to provide involuntary treatment. Where a patient refuses treatment without decision-making capacity, anticipated harms must be ‘serious’, but only so serious as to justify overriding the patient’s refusal, taking into account the harms involuntary treatment itself may cause. In such cases, whether a person can be detained and treated will hinge largely on each jurisdiction’s least restrictive criterion.

## Introduction

Every Australian state and territory has mental health legislation that strictly limits the circumstances in which a person can be made subject to involuntary treatment (*Mental Health Act 2015* (ACT), *Mental Health and Related Services Act 1998* (NT), *Mental Health Act 2007* (NSW), *Mental Health Act 2016* (Qld), *Mental Health Act 2009* (SA), *Mental Health Act 2013* (Tas), *Mental Health and Wellbeing Act 2022* (Vic), *Mental Health Act 2014* (WA)). The criteria that must be met to permit involuntary treatment across the legislative schemes, are broadly similar. First, the person must have a mental illness or disorder, and the terms used are variously defined. Second, it must be judged that without treatment, the person or others may suffer a specified type or degree of harm – the details of which are outlined in the sections below. Third, it must be judged that involuntary treatment is the least restrictive way to prevent that harm. In the majority of Australian states there is a fourth criterion – that the person refusing treatment lacks decision-making capacity.

Lacking legal training, psychiatrists and psychiatric trainees are liable to misinterpret the ‘harm criterion’, at least as it applies to protecting patients from harm to themselves. If, the threshold for potential harm is imagined to be higher than the law actually requires, some people who may have benefitted from treatment may not receive it. Conversely, if it is imagined to be lower than that it is, some people may be subject to unlawful detention.

This paper surveys the meaning and clinical impact of the harm criteria across Australia’s jurisdictions.

## Methods

We reviewed the relevant provisions for involuntary inpatient treatment in each jurisdiction, read Court decisions making reference to those provisions or similar provisions in previous acts, sought out relevant explanatory memoranda and second reading speeches, and located advice published by governments on the interpretation of the provisions.

## Results

Table 1 sets out the various harm criteria as they appear in the different mental health acts across Australia. Though all the harm criteria are broadly similar, they differ in important ways.

**Table 1.** The harm criteria in Australian mental health legislation.

Jurisdiction	Provision	Part of	Harm criterion
*Criteria allowing involuntary treatment as a means of providing protection from ‘serious harm’ to the patient*			
New South Wales	s 14	Definition of a ‘mentally ill person’	With respect to mentally ill persons, owing to the person’s mental illness, ‘there are reasonable grounds for believing that care, treatment or control of the person is necessary: (a) for the person’s own protection from serious harm, or (b) for the protection of others from serious harm’. In considering this issue, ‘the continuing condition of the person, including any likely deterioration in the person’s condition and the likely effects of any such deterioration, are to be taken into account’.
Queensland	s 12(1)(c) [incorporating definition from s 9].	Treatment criteria	‘[B]ecause of the person’s illness, the absence of involuntary treatment, or the absence of continued involuntary treatment, is likely to result in – (i) imminent serious harm [including physical, psychological and emotional harm] to the person or others; or (ii) the person suffering serious mental or physical deterioration’.
Victoria	s 143(b)	Compulsory treatment criteria	‘[B]ecause the person has mental illness, the person needs immediate treatment to prevent – (i) serious deterioration in the person’s mental or physical health; or (ii) serious harm to the person or to another person’.
Western Australia	s 25(1)(b)(ii)	Criteria for involuntary treatment order	‘[B]ecause of the mental illness, there is – (i) a significant risk to the health or safety of the person or to the safety of another person; or (ii) a significant risk of serious harm to the person or to another person’
*Criteria allowing involuntary treatment as a means of providing protection from ‘physical or mental’ harm*			
South Australia	s 21(1)(b)	Criteria to make a level 1 inpatient treatment order	‘[B]ecause of the mental illness, the person requires treatment for the person’s own protection from harm (whether physical or mental, and including harm involved in the continuation or deterioration of the person’s condition) or for the protection of others from harm’
*Criteria allowing involuntary as a means of providing protection from harm or risk to the patient’s ‘health or safety’*			
Tasmania	s 40(b)	Treatment criteria	‘[W]ithout treatment, the mental illness will, or is likely to, seriously harm – (i) the person’s health or safety; or (ii) the safety of other persons’.
Western Australia	s 25(1)(b)(i)	Criteria for involuntary treatment order	‘[B]ecause of the mental illness, there is – (i) a significant risk to the health or safety of the person or to the safety of another person; or (ii) a significant risk of serious harm to the person or to another person’
*Criteria allowing involuntary treatment as a means of providing protection from patients doing or causing serious harm to themselves*			
Australian Capital Territory	s 58(2)(c), (d)	Psychiatric treatment order criteria	‘[B]ecause of the mental illness, the person – (i) is doing, or is likely to do, serious harm to themself or someone else; or (ii) is suffering, or is likely to suffer, serious mental or physical deterioration’. In cases where a person with decision-making capacity refuses to consent, ‘the harm or deterioration, or likely harm or deterioration, . . . is of such a serious nature that it outweighs the person’s right to refuse to consent’.
Northern Territory	s 14(b)(ii) [incorporating definition from s 4].	Criteria for involuntary admission on the grounds of mental illness	‘[W]ithout the treatment, the person is likely to: (A) cause serious harm [including financial harm and loss of reputation] to himself or herself or to someone else; or (B) suffer serious mental or physical deterioration’.
	s 15(c) [incorporating definition from s 4].	Criteria for involuntary admission on the grounds of mental disturbance	[U]nless the person receives treatment and care at an approved treatment facility, he or she: (i) is likely to cause serious harm [including financial harm and loss of reputation] to himself or herself or to someone else; or (ii) will represent a substantial danger to the general community; or (iii) is likely to suffer serious mental or physical deterioration.
*Criteria allowing involuntary treatment as a means of providing protection from ‘serious physical harm’ to the patient*			
New South Wales	s 15	Definition of a ‘mentally disordered person’	With respect mentally disordered persons, ‘the person’s behaviour for the time being is so irrational as to justify a conclusion on reasonable grounds that temporary care, treatment or control of the person is necessary – (a) for the person’s own protection from serious physical harm, or (b) for the protection of others from serious physical harm’.

In order to grasp the meaning and clinical impact of the various harm criteria, it is important to examine three aspects of each. These are:

1. the breadth of scope of the harms envisaged;
2. how severe any harm must to be to trigger the criterion;
3. how likely it must be that the envisaged harm will arise.

Construing the meaning of any legislative provision requires consideration of the ‘ordinary and natural meaning’ of the words taking into account context and purpose of the legislation (*Amalgamated Society of Engineers v Adelaide Steamship Co Ltd* (1920) 28 CLR 129, *Alcan (NT) Alumina Pty Ltd v Commissioner of Territory Revenue* [2009] HCA 41, *Australian Education Union v Department of Education and Children’s Services* [2012] HCA 3).

### The breadth of the harms envisaged

#### New South Wales, Queensland, Victoria and Western Australia

In Queensland, Victoria and Western Australia (WA), the harm criterion refers to ‘serious harm’ (see Table 1). ‘Serious harm’ is also required to detain a person with a ‘mental illness’, but not ‘mentally disordered persons’ (as each term is defined) in New South Wales (NSW) (*Mental Health Act 2007* (NSW), ss 4,14,15).

The only court case we have been able to locate that is directly relevant to the breadth of a harm criterion as currently expressed in any of these jurisdictions is a 2011 NSW Supreme Court case – *Re J (No. 2)* [2011] NSWSC 1224 (‘*Re J*’). This case concerned a man with mania who appealed against the decision of the NSW Mental Health Review Tribunal (MHRT) that he remain an involuntary patient in Sydney’s Prince of Wales Hospital. J had recently received a very large payout on his life insurance and superannuation and, by time the matter reached the Court, the purported basis for J’s continued detention was a concern that, owing to his mania, he might dissipate his money unwisely.

Commenting on the breadth of harms that might be considered relevant to ongoing detention, White J said in *obiter dicta*, ‘I think there would be much to be said for the submission of counsel for the plaintiff that serious harm under s 14 refers to what counsel calls either physical harm or psychological harm’ (*Re J*, [93]). Furthermore, His Honour said that he would accept that the section ‘would permit the continued involuntary detention of a person suffering from mental illness if that were necessary to protect the person from serious harm, being the harm associated with the illness itself’ (*Re J*, [101]).

This notion that the harms of concern likely include harms associated with the illness itself appears to be under appreciated in NSW. In cases of severe depression, for example, clinicians are often focused on the possibility of future self-harm or suicide, overlooking the serious despair, distress or guilt that is often part and parcel of the illness itself. While persecutory delusions might cause people to harm themselves or others, they are usually terrifying, and that terror is a harm associated with the illness itself. Similarly, while not everyone who experiences hallucinations perceives them as harmful, for many people hallucinations are very frightening and might be reasonably regarded as seriously harming the person.

The breadth of the harms intended by the NSW harm criteria was further reinforced when, following a 2014 NSW Coroner’s inquiry, where concerns were expressed about psychiatrists interpreting the harm criterion too narrowly, the NSW Chief Psychiatrist issued a communique stating that the ‘serious harm’ referred to in the NSW Act, was intended to be a broad concept that may include, not only physical harm, but ‘emotional/psychological harm’, neglect of self, harm to a person’s relationships, and financial and reputational harm (*Inquest into the deaths of Nicholas Waterlow and Chloe Heuston – Reasons for Findings (2009/473553)* [2014] NSWCorC 2 (10 January 2014), New South Wales Health, 2015; Ryan et al., 2012).

Interestingly with respect to the possible inclusion of financial and reputational harm, in *Re J*, White J suggested that an interpretation that these harms were among the relevant harms would require consideration of NSW legislative history in construing the relevant section and that such an approach may not be appropriate (*Re J*, [88-95]). This casts doubt on whether financial and reputational harms alone, *are* among the types of harms that may be considered by clinicians as a basis for involuntary treatment, in NSW at least.

Even if financial and reputational harms are not relevant on their own, in some circumstances it may be that protecting a person from a severe financial harm might also protect them from a consequent serious mental (or even physical) harm. Where that is the case, the provisions may be engaged by that route.

In neither Victoria nor WA is ‘harm’ defined within the legislation, however in Queensland, the Act explicitly defines ‘harm’ to include ‘physical, psychological and emotional harm’ (*Mental Health Act 2016* (Qld), s 9) Financial and reputational harm are not explicitly referred to in any of the four acts.

In WA the *Mental Health Act* requires that decisions around the need for an inpatient order be made having regard to guidelines published under section 547 of the Act (*Mental Health Act 2014* (WA), ss 25, 547) These guidelines provide a non-exhaustive list of examples of ‘risks to self’, presumably intended to be examples of ‘harm’ to self. These examples largely overlap with the NSW Chief Psychiatrist’s examples, including references to financial and reputational harm, but they add ‘absconding and wandering’, ‘drug (illicit and prescribed) and alcohol intoxication, misuse or withdrawal’, ‘lack of recognition and treatment for’ medical conditions, and risk to dignity and social status (Chief Psychiatrist of Western Australia, 2015: 14). The guidelines also point to potential harms from others including: physical or sexual abuse or assault; emotional harm or abuse; harassment; financial abuse; and, neglect. The breadth of relevant harms implied by the listed examples would likely be given particular weight in a WA court, owing to the legislative foundation of the guideline.

The legislative provisions of all four jurisdictions require that the clinician consider, not only any harm that might be relevant at the time of the review, but also the possibility of the person’s condition deteriorating and the consequences of such a deterioration.

This requirement to consider the patient’s future is particularly important in Queensland as, unique among Australian jurisdictions, one subsection of the harm criterion provision refers to protecting the individual from ‘*imminent* serious harm’ [emphasis added]. This language appears to truncate the horizon of concern that Queensland clinicians may have regarding potential harms. However, the Act also permits involuntary treatment to protect a person from ‘serious mental or physical deterioration’ which may lengthen the timeframe clinicians are able to consider. Nevertheless, the deterioration provision only relates to ‘mental’ or ‘physical’ deterioration, so the prospect of financial and reputational harms would only be relevant to the extent that they are clearly linked with mental or physical deterioration.

In WA deterioration is not explicitly referred to, however, some consideration of the patient’s future status appears to be included since the statute refers to ‘a significant *risk* of serious harm’ [emphasis added] and ‘risk’ is an inherently forward-looking concept.

#### South Australia

In South Australia (SA) an order may be made to protect a person from ‘physical or mental’ harm, including ‘harm involved in the continuation or deterioration of the person’s condition’. This might be thought by clinicians to exclude consideration of financial or reputational harms as stand-alone harms. Nevertheless, the South Australian Government’s *Clinician’s Guide & Code of Practice* provides an example where a person with mania ‘decides impulsively to give their life savings to a charity leaving them with no income at all’ and suggests that ‘this behaviour could be interpreted as posing harm to the person’s wellbeing’ and therefore could meet the harm criterion (Government of South Australia, 2009: 58). The *Guide* suggests that reference to ‘deterioration of the person’s condition’ in the Act, might be thought of as deterioration in ‘wellbeing’. While the authors do not believe it is clear that such an example would necessarily be found by a Court to fall within the meaning of the provisions, it would be arguable where financial harm would also entail severe physical or mental harm.

#### Tasmania

The relevant Tasmanian criterion does not refer to ‘harm’ per se, but rather to serious harm to the ‘person’s health or safety’. Again, this wording appears to exclude financial or reputational harms as stand-alone harms.

The 2020 Tasmanian Supreme Court case, *B v Mental Health Tribunal* [2020] TASSC 10 (*B v MHT*), concerned a woman with schizophrenia who appealed against the decision made by the State’s Mental Health Tribunal (MHT) for her continued detention as an involuntary patient. Blows CJ, accepted without issue that the Tribunal’s view that an ‘exacerbation’ of suicidal ideation and paranoid ideas that had led the patient to become ‘distraught and tortured’ posed a serious harm to her health or safety (*B v MHT*, [8]). This indicates that in Tasmania, like NSW, harms associated with the illness itself are likely to engage the harm criterion, provided they are ‘serious’.

With respect to future harms, the Tasmanian legislation refers to harms ‘likely’ arising. We discuss the assessment of ‘likelihood’ of future harms below.

#### The ACT and Northern Territory

In the ACT, the harm criterion is engaged if it is felt that the person ‘is doing, or is likely to do’ serious harm to themself or someone else. Similarly, in the NT it is engaged if it is felt that the person is likely to ‘cause’, serious harm to themself or someone else.

These provisions refer to harm caused by the person’s own actions, which may also include harms due to self-neglect or other actions, in addition to frank self-harm. In both jurisdictions, the clinician must also consider ‘serious mental or physical deterioration’ whether or not the person is the ‘cause’ of that deterioration or its consequences. This language suggests that a range of potential harms may be considered similar in breadth to the psychological and physical harms relevant in New South Wales, Queensland and Victoria.

In the NT Act, ‘harm’ includes ‘financial harm and loss of reputation’ (*Mental Health and Related Services Act 1998* (NT), s4). As such it is the only Australian jurisdiction where financial and reputational harms are expressly within the scope of the harms of concern. There is no definition of harm in the ACT Act.

#### Mentally disordered patients in NSW

In NSW, it is possible to detain a person who does not suffer a ‘mental illness’, but who is a ‘mentally disordered person’, because their behaviour is ‘so irrational as to justify a conclusion on reasonable grounds that temporary care, treatment or control of the person is necessary . . . for the person’s own protection from serious *physical* harm’ [emphasis added] (*Mental Health Act 2007* (NSW), s 15) This provision, first introduced with the 1990 Act (*Mental Health Act 1990* (NSW), s 10) was intended for people who, because of an acute stressor were ‘overwhelmed by emotional turmoil’, ‘unable to control their actions and emotions’ and briefly ‘suicidal, or otherwise seriously out of control’ (Collins, 1990: 889).

Having substituted ‘irrational behaviour’ for ‘mental illness’ in the mentally disordered provisions, the NSW Parliament constrained the relevant harm criterion to the serious *physical* harms. As a result, a person may not be detained under this provision because of any of the wider psychological, relationship or other harms that engage the s 14 provision. It is worth noting now, in relation to the impact of a loss of decision-making capacity on the harm criterion as discussed below, that even if a person were behaving irrationally in the way s 15 envisages, that would not, on its own, indicate that the person was incompetently refusing admission (Ryan et al., 2015).

### The severity of the harms envisaged

In every jurisdiction except SA and WA all the relevant harms are proceeded by the word ‘serious’. ‘Serious’ also qualifies the envisaged harm arising in sub 25(1)(b)(ii) in the WA legislation, though it does not appear in the phrasing of sub 25(1)(b)(i), which reads ‘a significant risk to the health or safety of the person’.

In all the provisions where the word ‘serious’ occurs it acts as a qualifier to the types of harm that ought to be considered.

‘Serious’ is not defined in any of the acts. It is not a particularly precise term, but it is likely that a court would interpret it in the context of the objects and principles of the relevant legislation. Each act makes prominent reference to protecting the rights of individuals who might be made subject to involuntary treatment, so the seriousness of the harms envisaged to be avoided would have to be proportionate to the deprivation of any rights that involuntary detention and treatment would manifest.

We have not found a court case that was required to adjudicate the issue of the ‘seriousness’ of envisaged harms specifically, however in 2021 the Full Court of the Supreme Court of Tasmania heard an appeal to one aspect of the decision in *B v MHT* in *CMB v Mental Health Tribunal* [2021] TASFC 4 (*CMB*). The contested aspect was not the meaning of the word ‘serious’, but the meaning of ‘likely’ (see below). Importantly for this discussion though, in determining the meaning of that term the justices took careful account of the Tasmanian Act’s objects, the ‘Mental health service delivery principles’ (*Mental Health Act* 2013 (Tas), ss 12, 15) and the degree to which CMB’s rights were being engaged by involuntary treatment.

It is likely that even without the express requirement that risk of harm be ‘serious’ in the SA and WA Acts, courts would similarly interpret these provisions as requiring the presence or likelihood of harms that are serious enough to be proportionate to the loss of civil rights the patient will endure as a consequence of involuntary treatment.

In the jurisdictions that still permit a competent refusal of treatment to be overridden in certain circumstances – ACT, NT, NSW and Victoria – compulsory detention and treatment directly engage basic common law rights of freedom of movement and bodily integrity. That is, the rights not to be confined and not to be physically interfered with without consent. In such circumstances, a court would be likely to require that any potential harm justifying the infringement of these rights be very grave indeed. Even in jurisdictions without an express capacity requirement, a patient’s decision-making capacity is therefore relevant to judging the seriousness of any contemplated harm.

Relevantly, in *CMB*, the recognition that the patient lacked decision-making capacity was a prominent element of the reasoning in the Full Court’s determination of the meaning of ‘likely’.

### The likelihood of the envisaged harms arising

With the possible exception of WA, no jurisdiction’s legislative provisions explicitly require a structured risk assessment to determine the likelihood of serious harm. Nonetheless, some clinicians and tribunals may frame their judgements in risk-assessment terms, and expert evidence is often presented this way. This is noteworthy because, although it is now widely accepted that no demographic or clinical factors can usefully stratify patients by their likelihood of suicide or serious self-harm, many clinicians still assume the legislation demands such an approach (Large et al., 2017).

#### The ACT, Queensland and Tasmania

To justify involuntary treatment in the ACT, Queensland and Tasmania clinicians must come to the view that without treatment, it is ‘likely’ that the serious harms will arise. In addition, in NSW, clinicians considering whether a person needs protection from serious harm, must take into account ‘any *likely* deterioration in the person’s condition and the *likely* effects of any such deterioration’ [emphasis added] (*Mental Health Act 2007* (NSW), s 14).

Like ‘serious’, the word ‘likely’ is open to a number of possible interpretations. The meaning of ‘likely’ was one of the contested issues in *B v MHT*, and, as noted above, it was interrogated further on appeal in *CMB*. In the first instance case, having had regard to the purpose and context of the provision and having rejected other possible meanings such as ‘more probable than not’ and ‘a possibility that is more than remote or theoretical possibility’, Blows CJ had found that the term should be interpreted as referring to ‘something that might well happen’ (*B v MHT*, [14]). On appeal, all three justices of the Full Court agreed.

While courts in other jurisdictions are not bound to apply the same interpretation to ‘likely’, a unanimous decision of the Tasmanian appeal court is persuasive precedent for other jurisdictions.

#### The NT

Like clinicians in the ACT, Queensland and Tasmania, to justify involuntary treatment in the NT, clinicians must come to the view that without treatment it ‘is likely’ that the serious harms of concern will arise.

The meaning of ‘is likely’ in the Northern Territory provisions was the subject of the NT Supreme Court case, *KMD v the Mental Health Review Tribunal & Anor* [2020] NTSC 13 (*KMD*). *KMD* concerned a woman with a ‘serious delusional disorder’ who, in May 2013, under the influence of the delusion that ‘her son was being sexually assaulted and was in danger of further sexual assault’, had committed eight serious offences including attempted murder. At the time that *KMD* was being heard, the woman had been found not guilty by reason of mental impairment and a custodial term imposed. Her delusions had persisted and, in the face of her incompetent refusal of treatment in a custodial setting, she was transferred involuntarily to a psychiatry unit so that she might be provided involuntary treatment. KMD appealed against the involuntary treatment order imposed by the NT MHRT on the ground (among others) that she was not ‘likely’ to harm herself or others while in custody. Allowing the appeal, and without needing to determine the exact meaning of ‘is likely’, Barr J found that the provision required ‘a realistic consideration of a person’s overall situation’, and that since there had been no indication that KMD ‘was likely to cause harm – serious or otherwise – to any person within the custodial environment’ (*KMD*, [31], [42]), and that she could not be released from custody without Supreme Court intervention, it could not be said that without treatment she was likely to cause anyone any serious harm. (The possibility that the presence of KMD’s presumably very distressing delusions could by itself constitute ‘serious harm’ was not argued).

#### NSW, South Australia and Victoria

In NSW, SA and Victoria, an involuntary treatment order may be made if the clinician comes to the view that the order is necessary for the person’s protection (or for other’s protection) from the relevant envisaged harms. The wording of each provision does not explicitly require the formation of a view on the *likelihood* of the envisaged harms. However, it seems likely that there would be an expectation that in order to form the view that protection against the harm was needed, a clinician would have to form the view that such a harm was, at least, ‘something that might well happen’ in situations where a refusal of treatment lacked decision-making capacity.

In the ACT, NSW and Victoria, in cases where a treatment order was applied for despite a person’s competent refusal, it may be that the likelihood of the envisaged harm would need to be considered in conjunction with the seriousness of the harm envisaged so that involuntary detention and treatment would be proportionate to the consequent deprivation of the person’s rights.

#### Western Australia

In WA, the statutory question is whether there is a ‘significant risk’ of serious harm. The 2015 WA Chief Psychiatrist’s Guidelines, which remain in force, suggest that clinicians should use standardised risk-assessment tools, while noting that ‘actuarial risk assessment tools are of limited predictive value on their own’ (Chief Psychiatrist of Western Australia, 2015: para 4.2). Since 2015, it has become widely accepted that stratification by likelihood of serious harm is not a valid or useful way to determine who will most benefit from treatment. For clinicians and tribunals, the more relevant enquiry is whether, in an individual case, effective treatment is available and the statutory threshold of significant risk of serious harm is met.

## Discussion

The paucity of judicial interpretation of the differing harm criteria across the eight jurisdictions represents a significant limitation to our analysis. As we have shown, terms like ‘harm’, ‘serious’ and ‘likely’ are open to multiple interpretations and courts will construe them in the context of the facts of any case brought before them. Moreover, a court in one jurisdiction is not obliged to interpret terms in the same way as those in other jurisdictions, though such interpretations may be ‘persuasive’. Naturally broad terms such as ‘serious harm’ or ‘likely’ envisage the exercise of clinical discretion in individual cases, and the effect in practice is that courts often heavily rely on clinical judgement in applying these provisions.

Despite these limitations, it is possible for clinicians to have a good general sense of the provisions they are required to work within. For example, in NSW the requirement that involuntary treatment must be ‘necessary . . . for the person’s own protection from serious harm’ is not expressly restricted to self-harm or suicide risk. Rather, an order for treatment may be given where it is necessary to protect a person from any serious harm – including any harms arising a result of the illness itself. This could encompass a harmful deterioration in the person’s condition or serious distress. Furthermore, this understanding is important if people in need are not to be unnecessarily deprived of treatment, as may occur if a clinician mistakenly believes that a mentally ill person must be judged ‘more likely than not’ to seriously harm or kill themselves (or others), in order to receive treatment.

Though doctors may find the term ‘serious harm’ unfamiliar or even concerning, it should not dissuade them from ordinary responsible clinical decision-making in day-to-day practice in cases where a patient, who lacks decision-making capacity due to mental illness, refuses treatment. In such circumstances, a responsible clinician would not consider detaining a person in hospital involuntarily because of trivial risks or far-fetched possibilities – or anything less than likely serious harms.

Leaving the NSW mentally disordered criteria to one side, as general rule, it is most likely that the harm criterion in each of the various mental health acts requires clinicians to consider serious harms in a wide range of domains that ‘might well happen’ without treatment, considering the patient’s individual circumstances. The relevant harms likely include the psychological harms of the illness itself. Financial and reputational harm are definitely included in the NT, but beyond the NT the inclusion of those harms is less clear, unless perhaps the financial or reputational harms were so severe as to engender serious psychological harm. However, financial harms may often be avoided by financial orders that are less restrictive than inpatient treatment orders (see *Re J*) and almost all behaviours that raise concerns about serious reputational harm also raise concerns about serious physical or psychological harms that would meet criteria without regard to reputational harms.

Though this review has focused primarily on criteria regarding harm to *self*, the protection from harm criteria in all jurisdictions also make reference to potential harm *to others*. In this context, it is worth observing that, in our society, if a person seriously physically harms another person, that action would almost invariably entail serious harm to the person themselves via the action’s legal and/or emotional consequences. Relevantly, in *KMD*, Barr J accepted in *obiter dictum* that ‘the significant detriments of ongoing incarceration are properly characterised as ‘serious harm’’ in the NT legislation (*KMD*, [47]). This line of reasoning suggests that in all the provisions reviewed above, except arguably the NSW mentally disordered provisions, the concerns about serious physical harm to others might also engage concerns about serious harm to self.

## Conclusion

Assuming the psychological harms of the illness itself may be counted among the serious harms of concern in applying the various harm criteria, during most clinical encounters where involuntary hospitalisation is being considered, it will not be necessary for clinicians to auger about future possible serious harms, because serious psychological harms and serious harms to relationships will be evident there and then in the consultation room. Serious psychological harms due directly to the impact of the illness will, if present, usually be evident in the mental state examination. In addition, patients or their loved ones will report the serious harm that the illness is doing, at that time, to their relationships.

At the coalface, where a person is usually brought to the emergency department because they have recently overdosed, or have expressed suicidal ideation, or is otherwise seriously mentally ill, the harm criterion is likely to be the one of the three or four involuntary treatment criteria that is most easily met. Almost everyone in that circumstance might well, without treatment, suffer serious harm (broadly defined). In most clinical circumstances where involuntary treatment is being considered, the only thing that the harm criterion does, is set a floor price on the harms that clinicians must envisage a patient experiencing before they have legal warrant to provide involuntary treatment.

In cases where a patient’s refusal of treatment is made without decision-making capacity, those harms must still be serious, but only so serious as to justify overriding the incompetent refusal of treatment and account for any harms that involuntary treatment itself is likely to bring (Large et al., 2013). Although we have not been able to find any cases or official advice referring to the harms of involuntary treatment itself, in our view, consideration of compulsory treatment, even for people who lack decision-making capacity, must include some weighing of harms that the treatment itself is likely to bring.

Where a patient refuses treatment with decision-making capacity, involuntary treatment is not available in Queensland, SA, Tasmania or WA. In the other jurisdictions, clinicians must judge that the harms to be avoided are so serious as to outweigh the person’s right to refuse treatment and the harms likely to flow from overriding that refusal. Outside the ACT, legislation does not specify the circumstances in which competent refusals may be overridden, but such cases appear intended to be rare and exceptional. Even if one accepts that compulsory treatment could in theory be justified on the basis of self-harm, the envisaged harm would have to be very grave and its likelihood considerably greater than a possibility that ‘might well happen’.

In practice, when involuntary treatment is being contemplated for people whose refusal of treatment lacks capacity, the protection from harm criterion is likely to be met in most cases. For this reason, in these cases, whether or not a person can be involuntarily detained or treated will hinge almost entirely on each jurisdiction’s least restrictive criterion. Of all the treatment criteria, this will be the criterion, upon which most decisions will turn, and is the one that clinicians should most carefully interrogate with the patient and the patient’s family and loved ones, before reaching a conclusion that involuntary treatment is justified.
